# Seasonal fluctuations and diversity of Ingoldian mycobiota in two water bodies receiving different effluents at Assiut Governorate (Upper Egypt)

**DOI:** 10.1186/s12866-023-02903-z

**Published:** 2023-06-06

**Authors:** Abdel-Raouf M. Khallil, Essam H. Ali, Sabreen S. Ibrahim, Elhagag Ahmed Hassan

**Affiliations:** Botany and Microbiology Department, Faculty of Science, University of Assiut, Assiut, EG-71515 Egypt

**Keywords:** Aquatic hyphomycetes, Fungal biodiversity, Sewage and industrial effluents, Xenobiotic compounds

## Abstract

In the current study, fifty-eight Ingoldain fungal species assignable to forty-one genera were recovered from two water bodies receiving the treated sewage and the effluents of oils and soaps factory at Assiut Governorate (Upper Egypt), of which *Anguillospora*, *Amniculicola*, *Flagellospora*, and *Mycocentrospora* were the most prevalent genera. The most widespread identified species were *Anguillospora furtive, Amniculicola longissima* and* Flagellospora fusarioides*. Forty-three species were identified for the first time in Egypt. The most Ingoldain taxa were estimated for El-Zinnar canal, with the highest recorded taxa in winter. Whereas, the highest dominance of Ingoldian fungi was estimated for the El-Ibrahimia canal. The highest Simpson and Shannon diversity indexes were estimated for El-Zinnar canal samples recording 0.9683 and 3.741, respectively. The poorest water sites with Ingoldian fungi were those exposed directly to either treated sewage or industrial effluents, with which relatively higher values of water conductivity, cations and anions. Water temperature was the main abiotic factor driving the seasonal occurrence of Ingoldian fungi. It is interesting to isolate some Ingoldian fungal species from the stressful water sites receiving the effluents which provide valuable insights regarding their adaptation, predictive and putative role as bioindicators and their potentiality in pollutants degradation, organic decomposition, and transformation of xenobiotic compounds.

## Introduction

Aquatic Hyphomycetes [1], also named Ingoldian fungi [2] constitute a group of anamorphic fungi of ascomycetes or basidiomycetes [3] adapted to aquatic environments and are typically producing relatively large multiradiate (often tetraradiate), sigmoid or spherical conidia, whose tips may be covered with sticky mucilage [4], on submerged and deciduous plant debris (leaf litter, petioles, bark, etc.). Ingoldian fungi produce conidia exclusively in the aquatic environment or the interstitial water among soil particles [5]. Ingoldian fungi play pivotal roles in the biological processes of many ecosystems and nutrient recycling [6]. They colonize allochthonous and autochthonous organic matter in streams and rivers and initiate their degradation to make it more palatable and nutritious food source to aquatic invertebrate consumers [7]. Human enterprises such as industry, urbanization and agriculture greatly impact rivers and streams, and consequently pristine ecosystems are becoming scarce. Various effluents are increasingly being considered not only for the more feasible use in irrigation but also to produce quality water for urban and drinking [8]. Ingoldian fungi are generally associated with clean and well-aerated freshwaters and are believed to be sensitive to pollution [9]. A decline in aquatic hyphomycetes diversity has been found in streams affected by organic pollution [10] or heavy metals [11]. However, the occurrence of several aquatic hyphomycete species in heavily polluted streams has been reported [12–14]. So, the presence of aquatic hyphomycetes is not limited to clean water habitats, they are also present and active in the degradation of organic material in harsh habitats such as aquatic ecosystems that are contaminated by heavy metals and xenobiotic compounds [15]. The disposal of untreated or imperfect treated effluents can impact microbial diversity of natural environments, consequently affecting the functioning and health of aquatic environments [16, 17]. In the endeavor to unveil the global distribution patterns of Ingoldian fungi occurring in various water areas, several studies have been conducted worldwide [18–22]. Nevertheless, Cudowski et al*.* [23] stated that existing knowledge on aquatic fungi is fragmentary and it is estimated that only approximately 7% of the total number of species of aquatic fungi have been identified and described to date. This may be ascribed to the fact that taxonomical studies are usually conducted using only microscopic methods [24], which are time consuming and only allow the identification of fungi to the rank of genus with high accuracy, while species designations are more problematic. Despite the widespread and abundance of the Ingoldian fungi all over the world being observed from the Arctic Pole to Equator Line [2], the least studied regions are in Africa, with the exception of Nigeria [25], South Africa [26] and Libya [27] that have been largely neglected [28]. In Egypt, as a subtropical region and African country, knowledge concerning seasonality and diversity of Ingoldian fungi are in its infancy and were practically untouched until the pioneering work of El-Hissy et al*.* [29] and Khallil et al*.* [30]. Thus, the current investigation aims to shed the light and improve our understanding of the seasonal variation, biodiversity and occurrence of Ingoldian fungi in water bodies particularly those receiving the treated sewage and the effluents of oils and soaps factory at Assiut Governorate (Upper Egypt). In addition, the interaction between fungal occurrences and some abiotic factors of water were also considered.

## Materials and methods

### Description of study area

**Fig. 1:**
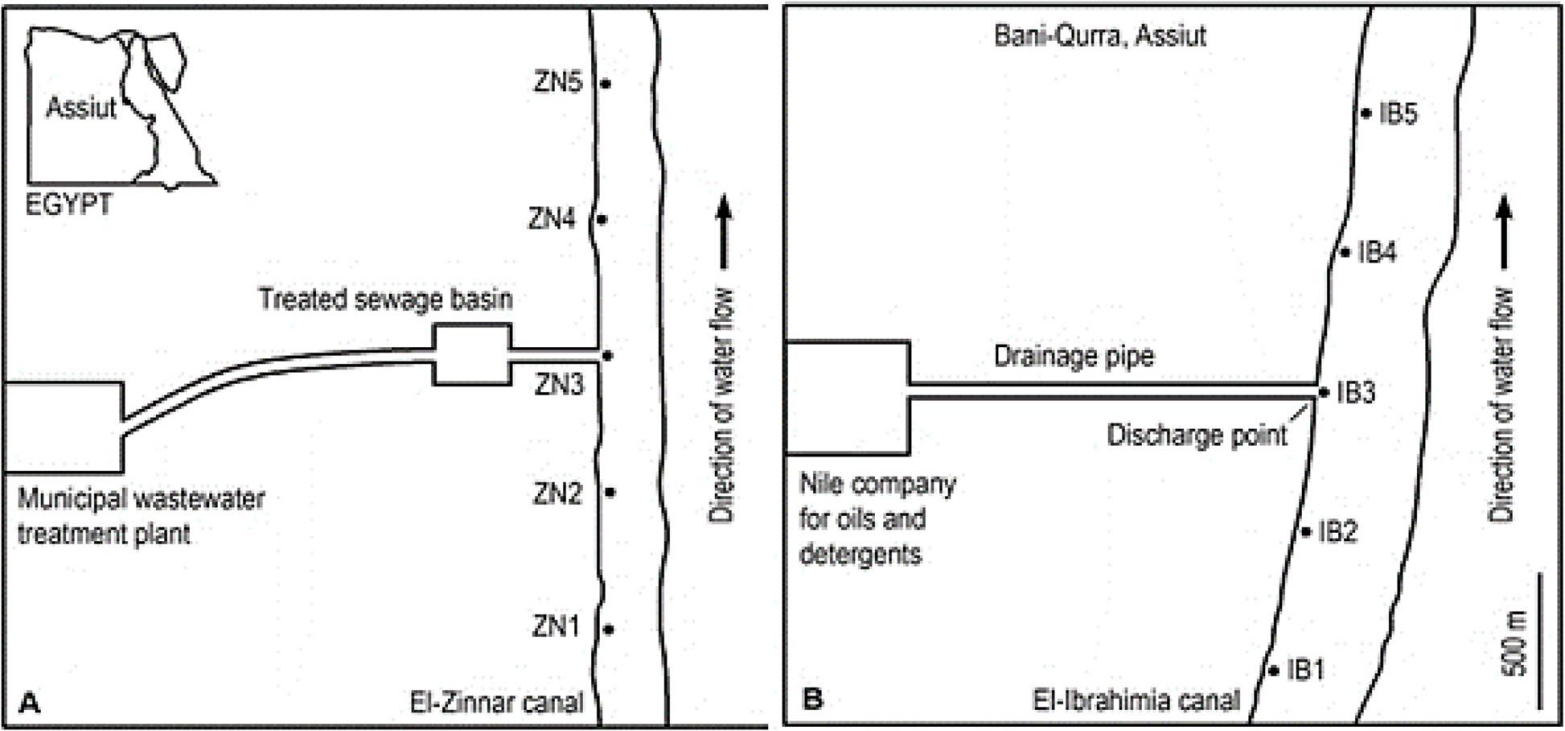
Diagrams illustrating the sampling sites on El-Zinnar irrigation canal (**A**) receiving treated sewage water (Arab El-Madabegh, Assiut Governorate) and the sampling sites on El-Ibrahimia irrigation canal (**B**) receiving industrial effluents of the oils and detergents factory (Nile Company, Bani-Qurra, Assiut Governorate)

**Fig. 2:**
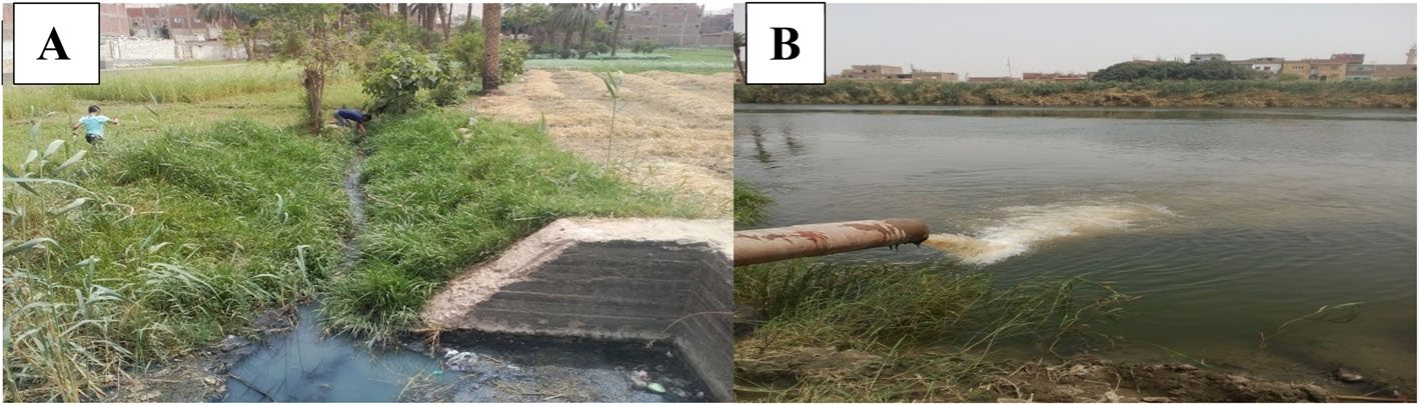
The sampling sites of El-Zinnar irrigation canal (**A**) that receive the treated sewage water (Arab El-Madabegh, Assiut Governorate) and the sampling sites of El-Ibrahimia irrigation canal (**B**) that receive the industrial effluents of Nile company of oils and detergents factory (Bani-Qurra, Assiut Governorate)

Egypt is located between latitude 22º and 31º north. The astronomical location of Egypt is subtropical region (Between latitude 18º, 30º north and south). The study areas were described elsewhere [31]. In the municipal plant (Arab El-Madabigh village; about 5 km north-west Assiut city, about 365 km south Cairo), the sewage wastewater is subjected to series of successive treatment stages (includes physical, chemical and biological treatments as well as sludge removal facilities), collected in a basin and eventually discharged into El-Zinnar canal which represents a significant water resource for irrigation. Five surface water samples were collected seasonally from five sites along about 2 km (at about 500 m intervals) of El-Zinnar canal (ZN_1_ – ZN_5_) as shown in (Figs. 1 and 2). The oils and detergents factory (Nile Company, Bani-Qurra) locates about 40 km north Assiut city (about 325 km south Cairo). The factory effluents are introduced directly into the main stream of the El-Ibrahimia irrigation canal (the biggest irrigation canal in Egypt which extends about 365 km and represents the main source for irrigation in middle Egypt Governorates). Five surface water samples were collected seasonally from five successive water sites (IB_1_- IB_5_) along about 2 km (at about 500 m intervals) of the El-Ibrahimia canal. IB_3_ directly receives the industrial effluents of the factory via discharge pipe (Figs. 1 and 2).

### Sampling program

#### Samples collection

Submerged mixed leaf litter samples and surface water samples were concomitantly collected seasonally from the different sites of the two described study areas (El-Zinnar canal and Ibrahimia canal) during winter 2017 to autumn 2018. Submerged plant litters were collected from the different water sites in polyethylene bags, brought to the laboratory and kept at 4 °C till fungal analysis. Water samples were also collected from the same sites using sterile bottles (one bottle 1 L capacity for physico-chemical analysis and another with 500 ml capacity for Ingoldian fungi analyses).

### Physico-chemical analysis of water samples

Some physico-chemical characteristic of water samples such as water temperature was measured in situ with field probes Thermometer (liquid mercury in glass tube), pH and water conductivity were measured ex-situ using a combined pH and conductivity meter (Jenway Model 3450 conductivity/pH meter). The organic matter contents were chemically estimated based on Jackson [32]. Sodium (Na^+^), potassium (K^+^) (by flame photometry), calcium (Ca^+2^), magnesium (Mg^+2^), chloride (Cl^−^), phosphate (PO_4_^−3^) and Nitrate (NO_3_^−^), sulphate (SO_4_^−2^), bicarbonate (HCO_3_^−^) were determined according to APHA [33].

### Fungal isolation

A preliminary experiment was conducted to choose the best baiting substratum for the recovery of Ingoldian fungi and among 5 examined leaves (*Eichhornia crassipes, Ficus retusa, Ficus carica, Vitis vinifera* and* Salix mucronate*), *Ficus retusa* leaves were the best substrate. The collected plant litter samples were processed using two methodological approaches (direct examination and submerged incubation). Leaves were directly examined for associated fungi usually on the leaves edge or on exposed veins in areas where decaying is occurring [34]. Also, submerged incubation following Bärlocher [35] was employed, some of the plant litters which did not show sporulating structures were vigorously washed with tap water to remove mud or other debris, then they were cut into segments of approximately 1 cm^2^ and placed in Petri dishes (5 segments for each) containing sterile distilled water. The Petri dishes were incubated at 20 ± 2 °C for 30 days during which the plant segments were examined on alternate days under a low power microscope (× 125) to detect the conidia of hyphomycetes sporulating on the surface [36, 37]. Simultaneously, water in the Petri dishes was replaced by fresh water to minimize the growth of bacteria and other organisms. This process was repeated up to 60 days and the fungi were identified on the basis of the morphology of their conidia. Regarding the collected water samples, two techniques were employed. A filtration technique where water samples were collected in polyethylene bottles (500 mL), were filtered through membrane filters (8 um pore size), the filters are immediately fixed and stained [38] with 1–2 mL of lactophenol-fucsine solution and sterilized water. Filter was scrubbed and washed, and the suspension was centrifuged, placed on a concave slide and the material was examined under a microscope to determine and detect the species composition of spores in the water. A bating technique was also employed, where aliquots of water samples (about 30 mL each) were poured in Petri dishes (3 Petri dishes for each sample) containing small sterile discs of leaves (*Ficus* leaves) as baits [20, 36]. All treated petri dishes were incubated at 20 ± 2 °C for 60 days during which the growing fungi were followed and identified. To induce sporulation, the hyphomycetes which failed to sporulate, 10 leaf discs were placed under aeration in Erlenmeyer flasks containing 40 mL of sterile deionized water for 48 ± 4 h at 18 ºC.

### Purification

To isolate species in pure culture, a piece of leaf bearing abundant conidia was placed in few drops of sterilized water in a covered watch glass. In the course of half a day a conidial suspension will probably have developed in the water (this can be checked under low power). Single spores or fungal spore masses that were identified using the inverted microscope were streaked on 2% PDA and a germinated spore was picked up with the help of sterilized needle under a stereoscope and cultured on PDA growth medium [39] and incubated at 20 ± 2 º C [36]. The pure cultures were maintained on slopes of agar medium and stored at 5 – 10 º C and sub-cultured every 1- 2 months.

### Identification and documentation

Slides for microscopical observation were prepared during the incubation period and were observed using an Olympus CX 41 optical microscope. The fungal taxa were mainly identified based on their unique characteristic conidial morphology as they produce conidiophores and conidiogenous cells according to the relevant taxonomical keys [1, 2, 9, 22, 36, 37]. Photographs were taken using an optical microscope coupled with a camera (Olympus SC30 U-TV1X-2, T2 Tokyo, Japan).

### Analysis of fungal diversity and their correlation with the abiotic factors

Aquatic hyphomycetes fungal diversity, Cluster analysis and Canonical correspondence analysis were estimated using PAleontological STatistics (PAST), Version 3.25, USA. In order to reveal the correlation of the determined abiotic factors with particular species occurrence, Canonical correspondence analysis (CCA) was used. Canonical correspondence analysis [40] is an analysis of a site/species matrix where each site has given values for one or more environmental variables. The ordination axes are linear combinations of the environmental variables.

### Results and discussion

#### Abiotic factors of the experimented water samples

The results of the physicochemical characteristics of the surface water samples collected from the two experimented water bodies are summarized in (Table 1). Water temperature, electrical conductivity, organic matter contents and major cations and anions (Na^+^, K^+^, Ca^2+^, Mg^2+^, Clˉ, HCO_3_ˉ, PO_4_^3−^, SO_4_^2−^ and NO_3_ˉ) were unevenly distributed and noticeably varied depending upon the water body, sampling site and season. The highest values of these parameters were frequently recorded in water samples which were collected from either the main effluent sources or from the water sites which are directly exposed to these effluents regardless to the sampling season or the experimented water body (e. g. the water site ZN_3_ which receives directly the treated sewage water in case of El-Zinnar canal, and the water site IB_3_ in case of El-Ibrahimia canal which receives the effluents of oils and detergents factory). It seems that most of these effluents were diluted at the other sites downstream and did not differ much.

**Table 1:** Lowest values (L), highest values (H) and means (M) of some physicochemical features of water samples collected from five sites in El-Zinnar canal (ZN) and five sites in El-Ibrahimia canal (IB) during four seasons (from winter 2017 to autumn 2018)

Seasons	Winter						Spring					
Water body	ZN			IB			ZN			IB		
Parameters	L	H	M	L	H	M	L	H	M	L	H	M
Temperature (˚C)	20.7	21.5	21.2	21.0	23.0	22.0	19.0	20.0	19.2	20.0	21.0	20.5
pH	8.1	8.5	8.3	8.7	9.0	8.9	8.4	8.7	8.6	8.4	8.6	8.5
Conductivity (µS/cm)	357.0	469.0	415.0	338.0	730.0	428.2	24.0	215.0	166.3	181.5	184.8	183.7
Organic matter(mg/L)	9.0	18.2	12.3	6.5	12.4	10.42	2.0	33.8	12.9	6.2	11.3	8.7
Na^+^ (mg/L)	38.9	51.0	43.5	39.0	92.0	50.9	43.3	52.0	47.6	34.1	58.7	43.4
K^+^ (mg/L)	2.2	14.9	6.3	4.0	4.8	4.4	6.9	27.0	13.9	5.1	218.0	52.7
Ca^2+^ (mg/L)	60.0	116.0	75.2	61.0	98.0	72.6	100.0	140.0	118.0	76.0	134.0	97.2
Mg^2+^ (mg/L)	12.2	77.8	57.4	34.0	60.8	48.1	36.5	61.0	50.6	48.6	69.3	57.6
Cl^−^ (mg/L)	105.0	155.8	134.8	96.3	168.0	118.7	175.0	350.0	235.6	140.0	248.0	181.0
HCO_3_ˉ (mg/L)	420.9	457.5	433.7	366.0	610.0	466.7	162.7	200.0	181.3	135.5	183.0	160.8
NO_3_^−^ (mg/L)	4.3	16.1	11.2	3.9	17.9	9.7	4.28	31.2	22.1	6.1	69.7	30.3
PO_4_^3−^(mg/L)	0.9	3.4	1.7	0.8	20.1	5.0	1.2	24.0	10.1	1.0	8.0	3.3
SO_4_^2−^(mg/L)	51.5	105.0	69.4	21.7	211.0	76.1	12.0	111.0	41.9	46.7	106.6	65.6
Seasons	Summer						Autumn					
Water body	ZN			IB			ZN			IB		
Parameters	L	H	M	L	H	M	L	H	M	L	H	M
Temperature (˚C)	33.0	33.0	33.0	32.8	33.1	32.9	31.4	32.5	32.1	31.8	32.8	32.3
pH	7.5	8.0	7.8	8.4	8.5	8.4	7.3	8.1	7.8	7.9	8.5	8.3
Conductivity (µS/cm)	323.0	441.0	353.0	300.0	315.0	305.4	334.0	647.0	429.4	327.0	485.0	363.8
Organic matter(mg/L)	3.8	71.0	26.9	15.0	33.0	22.8	7.9	24.5	15.8	2.9	18.7	9.2
Na^+^ (mg/L)	36.0	57.6	44.3	36.8	44.0	40.26	29.4	60.9	39.1	25.3	45.4	31.0
K^+^ (mg/L)	7.2	34.0	13.6	4.7	31.2	11.58	3.7	9.6	5.3	2.6	3.93	3.4
Ca^2+^ (mg/L)	52.0	60.0	55.6	42.0	62.0	50.4	60.0	88.0	72.3	66.7	112.0	91.8
Mg^2+^ (mg/L)	48.0	58.0	52.5	42.5	60.0	47.6	68.7	90.5	81.4	46.6	74.4	62.7
Cl^−^ (mg/L)	112.0	130.0	119.8	102.0	128	111.8	280.0	322.0	298.3	263.0	315.0	290.5
HCO_3_ˉ (mg/L)	234.0	263.0	246.4	154.0	220	172.34	103.7	158.6	129.9	97.6	128.1	116.5
NO_3_^−^ (mg/L)	9.2	67.9	26.6	3.7	9.2	6.36	25.1	35.5	29.8	24.5	38.0	31.1
PO_4_^3−^(mg/L)	0.3	2.6	1.5	0.2	36.0	10.84	13.4	26.9	19.7	18.9	36.7	23.4
SO_4_^2−^(mg/L)	4.5	53.3	18.2	49.7	87.0	71.0	142.3	194.8	162.8	136.9	183.6	153.5

### Diversity and occurrence of Ingoldian fungi

The obtained data (Tables 2 and 3) elucidate the diversity, seasonal fluctuations and occurrence frequency of aquatic hyphomycetes in the two interesting aquatic habitats (Fig. 1 A, B) at the Assiut Governorate (Upper Egypt). The presented data revealed that 56 fungal species belonging to 38 fungal genera in addition to 19 unknown taxa emerged from El-Zinnar irrigation canal, during four seasons (from winter 2017 to autumn 2018). Out of recovered taxa, 46 species in addition to 19 unknown conidia are new record in Egypt. Whereas, 36 fungal species related to 23 fungal genera were recovered from El-Ibrahimia canal. It is worth mentioning that one taxon related to aquatic ascomycetes emerged during this investigation. The stressful water sites can be regarded as polluted sites since they are exposed directly to both effluents and were the poorest with Ingoldian fungi irrespective of the sampling season and water body. The structure of the community of aquatic hyphomycetes was nearly similar between all sampling sites, except the polluted sites, in terms of the number of species and their abundance. The diversity of aquatic hyphomycetes fungal communities recovered from the El-Zinnar and El-Ibrahimia cana was calculated including fungal taxa (s) which indicates the number of isolated ingoldian fungal species from collected samples, dominance (d) determining the dominance of fungal taxa in specific site or season; simpson index illustrates the evenness of the aquatic hyphomycetes fungal community and Shannon index is the diversity index that estimates the number of fungal individuals taking in account the number of fungal taxa. So, the presented data in (Table 3) showed that the highest fungal taxa were recorded for El-Zinnar canal in which the highest fungal taxa were recorded in winter. Whereas, the highest fungal dominance was recorded for El-Ibrahimia canal. The highest Simpson and Shannon diversity indexes were estimated for El-Zinnar canal samples recording 0.9683 and 3.741, respectively. These diversity indexes illustrates that the highest evenness of fungal community in El-Zinnar canal compared with El-Ibrahimia canal.

**Table 2:** Seasonal variations and frequency of occurrence of Ingoldian fungi recovered from five sites in El-Zinnar canal (ZN) receiving the treated sewage water and five sites in El-Ibrahimia canal (IB) receiving the industrial effluents of oils and detergents factory during four seasons (from winter 2017 to autumn 2018)

Seasons	Winter		spring		summer		autumn		Total	
Water body	ZN	IB	ZN	IB	ZN	IB	ZN	IB	NIC	OR
*Amniculicola longissimia* Ingold	1	1	2	3	0	0	3	3	13	H
*Anguillospora*	2	1	3	4	0	0	3	3	16	H
*A. filiformis*^*a*^ Greath	1	0	1	3	0	0	0	1	6	M
*A. furtiva *^*a*^ (Descals) Webster & Descals	2	1	2	3	0	0	3	3	14	H
*A. fustiformis*^*a*^ Marvanová & Descals	0	0	0	1	0	0	0	1	2	R
*A. rosea*^*a*^ (Descals) Webster & Descals	0	0	0	2	0	0	0	0	2	R
*Aquanectria*	1	3	0	0	1	1	0	0	6	M
*A. penicillioides* (Ingold) Lombard & Crous	0	2	0	0	1	1	0	0	4	L
*A. submerse* (Hudson) Lombard & Crous	1	1	0	0	0	0	0	0	2	R
*Arbusculna moniliformis *^*a*^ Descals & Marvanová	1	0	0	0	0	0	0	0	1	R
*Arthobotrys oligosporus*^*a*^ Fresen	1	0	0	0	0	0	0	1	2	R
*Brachiosphaera tropicalis*^*a*^ Nawawi	0	0	0	0	0	0	1	0	1	R
*Calcarispora hiemalis*^*a*^ Marvanová & Marvan	0	0	1	1	0	0	0	0	2	R
*Condylospora gigantean*^*a*^ Nawawi & kuthub	0	0	0	1	0	0	0	0	1	R
*Curvularia pandanicola*^*a*^ Tibpromma & Hyde	0	0	1	0	0	0	0	1	2	R
*Cylindrocarpon aquaticum*^*a*^ Marvanová & Descals	0	0	0	0	0	2	0	0	2	R
*Cyrenella elegans*^*a*^ Goch	1	0	0	0	0	0	0	0	1	R
*Dactylella*^*a*^	0	1	2	0	0	0	0	1	4	L
*D. arnaudii*^*a*^ Yadav	0	0	1	0	0	0	0	1	2	R
*D. clavate*^*a*^ Chen et al	0	0	0	0	0	0	0	1	1	R
*D. coccinella*^*a*^ Ying Yang & Xing Z. Liu	0	0	1	0	0	0	0	0	1	R
*D. cylindrospora*^*a*^ Rubner	0	1	0	0	0	0	0	0	1	R
*Dendroclathra lignicola*^*a*^ Voglmayr	0	1	0	0	0	0	0	0	1	R
*Dendrospora erecta*^*a*^ Ingold	0	0	0	1	0	0	0	0	1	R
*Diplocladiella scalaroides*^*a*^ Arnaud	0	0	1	0	0	0	0	0	1	R
*Excipularia fusispora*^*a*^ Sacc	1	0	0	0	0	0	0	0	1	R
*Filosporella aquatica*^*a*^ Nawawi	0	0	0	1	0	0	0	0	1	R
*Flagellospora*	2	2	0	0	0	3	0	1	8	M
*F. curvula* Ingold	0	2	0	0	0	3	0	0	5	L
*F. fusarioides*^*a*^ Iqbal	2	1	0	0	0	3	0	1	7	M
*Glarea lozoyensis*^*a*^ Bills &Peláez	1	1	1	0	0	0	0	0	3	l
*Globoconidiopsis cerradensis*^*a*^ Sepứlveda, Pereia-Carv. & Dianese	0	0	0	0	1	0	0	0	1	R
*Guanomyces polythrix*^*a*^ González, Hanlin &Ulloa	1	0	0	0	0	0	0	0	1	R
*Hormiokrypsis libocedri*^*a*^ Bat. & Nascim	1	0	0	0	0	0	0	0	1	R
*Lemonniera*	0	1	2	0	0	0	0	1	4	L
*L. aquatica* De Wild	0	0	1	0	0	0	0	1	2	R
*L. centrosphaera*^*a*^ Marvanová	0	0	0	0	0	0	0	1	1	R
*L. cornuta* Ranzoni	0	0	1	0	0	0	0	0	1	R
*Leptodiscella brevicatenata*^*a*^ Madrid, Cano, Gené & Guarro	0	0	0	0	0	1	0	0	1	R
*Lunulospora curvula* Ingold	0	1	2	0	0	0	0	1	4	L
Seasons	Winter		spring		summer		autumn		Total Seasons	
Water body	ZN	IB	ZN	IB	ZN	IB body	ZN	IB	NIC	OR
*Mycocentrospora*^*a*^	1	2	1	2	0	2	0	0	8	M
*M. angulata*^*a*^ Iqbal	0	0	0	1	0	0	0	0	1	R
*M. Aquatica*^*a*^ Iqbal	1	1	1	0	0	0	0	0	3	L
*M. clavata*^*a*^ Iqbal	0	0	1	1	0	0	0	0	2	R
*Mycocentrospora sp. *^*a*^ Descals et al	0	1	0	0	0	2	0	0	3	L
*Pestalotiopsis guepinii*^*a*^ Steyaert	1	0	0	0	0	0	0	0	1	R
*Polycladium equiseti* Ingold	0	0	1	0	0	0	0	0	1	R
*Pseudoanguillospora stricta*^*a*^ Iqbal	0	0	0	0	0	0	0	1	1	R
*Pyramidospora casuarinae* Nilsson	1	0	0	0	1	0	0	0	2	R
*Sigmoidea aurantiaca*^*a*^ Descals	2	1	0	0	1	3	0	0	7	M
*Sirastachys phangngaensis *^*a*^ Tibpromma & Hyde	0	0	1	0	0	0	1	0	2	R
*Sympodioclathra globose*^*a*^ Voglmayr	0	1	0	0	0	0	0	0	1	R
*Tricladiopsis flagelliformis*^*a*^ Descals	1	0	0	0	0	0	0	0	1	R
*Tricladium gracile*^*a*^ Ingold	0	0	0	0	0	0	1	0	1	R
*Triscelophorus*	1	0	0	0	0	4	0	0	5	M
*T. acuminatus*^*a*^ Nawawi	1	0	0	0	0	4	0	0	5	M
*T. monosporus* Ingold	1	0	0	0	0	4	0	0	5	M
*Tumularia tuberculate*^*a*^ Descals & Marvanová	0	0	0	0	0	1	0	0	1	R
*Varicosporium*	0	0	0	1	0	0	1	1	3	L
*V. delicatum* Iqbal	0	0	0	0	0	0	1	0	1	R
*V. elodeae* Kegel	0	0	0	1	0	0	0	0	1	R
*V. giganteum* Crane	0	0	0	0	0	0	1	1	2	R
*Ypsilina graminea*^*a*^ Descals, Webster & Marvanová	1	0	0	0	0	0	0	0	1	R
*Zopfiella*	1	0	0	1	0	0	0	1	3	L
*Z. latipes* Malloch & Cain	1	0	0	0	0	0	0	0	1	R
*Z. longicaudata*^*a*^ Arx	0	0	0	1	0	0	0	1	2	R
Unknown 1	0	0	0	0	0	1	0	0	1	R
Unknown 2	1	1	0	0	0	0	0	1	3	L
Unknown 3	0	0	0	0	1	0	0	0	1	R
Unknown 4	0	0	0	0	1	0	0	0	1	R
Unknown 5	0	0	0	0	1	0	0	0	1	R
Unknown 6	0	0	0	0	1	0	0	0	1	R
Unknown 7	0	1	0	0	0	0	0	0	1	R
Unknown 8	0	1	1	0	1	0	0	0	3	L
Unknown 9	0	0	1	0	0	0	0	0	1	R
Unknown 10	0	0	1	1	0	1	0	0	3	L
Unknown 11	0	0	1	1	0	1	0	0	3	L
Unknown 12	0	0	1	1	1	1	0	0	4	L
Unknown 13	0	0	0	0	1	0	0	0	1	R
Unknown 14	0	0	0	0	1	0	0	0	1	R
Unknown 15	0	0	0	0	1	0	0	0	1	R
Unknown 16	0	0	0	1	1	0	1	1	4	L
Unknown 17	0	0	0	0	0	0	0	1	1	R
Unknown 18	0	0	0	0	0	0	0	1	1	R
Unknown 19	0	0	0	1	0	0	0	0	1	R

Occurrence Remarks:

H (High occurrence): › 25% of total samples. M (Moderate occurrence): 12%—› 25% of total samples

L (Low occurrence): 6%—› 12% of total samples. R (Rare occurrence): <6% of total samples

^a^Recorded for the first time in Egypt

**Table 3:** Biodiversity of aquatic hyphomycete communities recovered from five sites in El-Zinnar canal (ZN) receiving the treated sewage water and five sites in El-Ibrahimia canal (IB) receiving the industrial effluents of oils and detergents factory during four seasons (from winter 2017 to autumn 2018)

Seasons Water body	Winter		Spring		Summer		Autumn		ZN	IB
	**ZN**	**IB**	**ZN**	**IB**	**ZN**	**IB**	**ZN**	**IB**		
**Taxa (S)**	27	18	20	25	14	30	12	20	75	69
**Dominance (D)**	0.045	0.068	0.06	0.072	0.071	0.087	0.167	0.08	0.032	0.034
**Simpson (1-D)**	0.9547	0.9321	0.94	0.928	0.929	0.913	0.833	0.92	0.968	0.966
**Shannon (H)**	3.142	2.736	2.857	2.768	2.639	2.563	1.936	2.67	3.741	3.608
**Evenness e**^**H/S**^	0.964	0.964	0.967	0.885	1	0.865	0.866	0.90	0.826	0.785

**Fig. 3:**
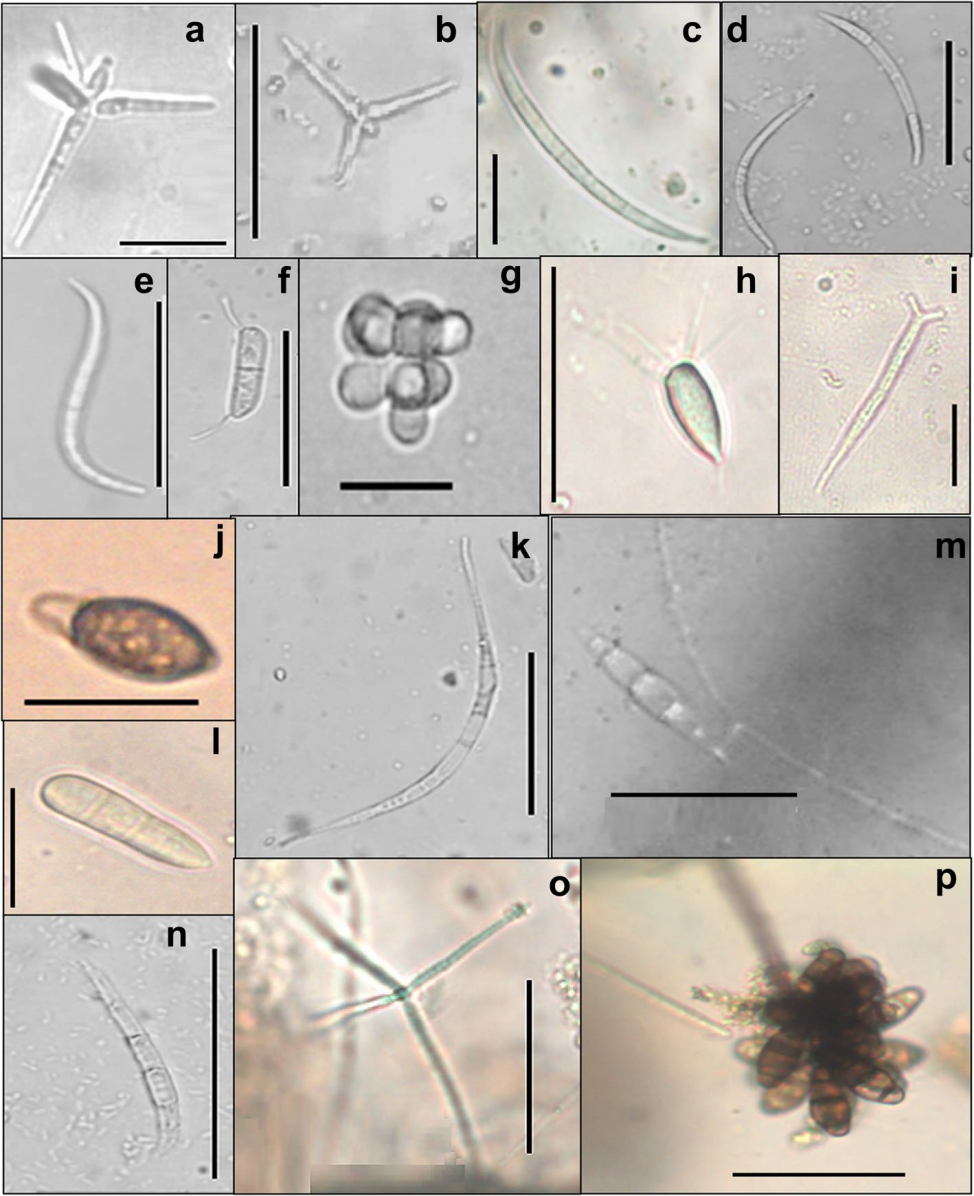
Micrographs of some isolated Aquatic Hyphomycetes were **a**: *Triscelophorus acuminatus*, **b**: *Triscelophorus monosporus*, **c**, **d**: *Flagellospora fusarioides*, **e**: *Aquanectria penicillioides*, **f**: *Leptodiscella brevicatenata*, **g**: *Pyramidospora casuarinae*, **h**: *Cyrenella elegans*, **i**: *Aquanectria submersa*, **j**: *Zopfiella latipes*, **k**: *Mycocentrospora* species, **l**: *Dactylella cylindrospora*, **m**: *Dactylella arnaudii*, **n**: *Sigmoidea aurantiaca*, **o**: *Lemonniera aquatica* and **p**: *Curvularia pandanicola*. All conidia with scale bar = 50 µm, except b with scale bar = 20 µm

The obtained results showed that fungal diversity, occurrence and species composition in water sites receiving municipally treated sewage water (El-Zinnar canal) and in sites receiving industrial effluents of oils and detergents factory (El-Ibrahimia canal) are different. Two genera (*Anguillospora* and *Amniculicola*) were the most widespread in both experimented water bodies but with the different occurrence and species diversity. *Anguillospora* was represented by 2 species (33.33% of total samples) in El-Zinnar canal whereas it as was represented by 4 species (40% of total samples) in El-Ibrahimial canal. *Amniculicola* was represented by only one species in each water body but with different ocurrence (25.00% and 40% of total samples in El-Zinnar canal and El-Ibrahimia canal, respectively). Similar variation patterns were recorded for several other genera and species (Table 2). *Lemonniera* (3 species) exhibited the broadest species spectrum in El-Zinnar canal while* Anguillospora* (4 species), *Mycocentrospora* (4 species) and *Dactylella* (3 species) exhibited the broadest species spectra in El-Ibrahimia canal. Both *Anguillospora furtive* and *Amniculicola longissima* were the predominant in both water bodies occurring in 29.16% and 25.00% of total samples in the El-Zinnar canal and El-Ibrahimia canal, respectively. *Flagellospora curvula* and* Flagellospora fusarioides* (25% of total samples each) were among the most widespread species in the El-Ibrahimia canal. Twenty identified taxa (*Arbusculna moniliformis*, *Brachiosphaera tropicalis*, *Cyrenella elegans*, *Dactylella coccinella*, *Diplocladiella scalaroides*, *Excipularia fusispora*, *Globoconidiopsis cerradensis*, *Guanomyces polythrix, Hormiokrypsis libocedri*, *Lemonniera aquatica*, *Lemonniera centrosphaera*, *Lemonniera cornuta*, *Pestalotiopsis guepinii*, *Polycladium equiseti*, *Pseudallescheria boydii*, *Pyramidospora casuarinae*, *Sirastachys phangngaensis, Tricladiopsis flagelliformis*, *Tricladium gracile*, *Varicosporium delicatum*, *Ypsilina graminea*, *Zopfiella latipes)* in addition to nine unknown taxa were recorded exclusively in El-Zinnar canal only and completely missed in El-Ibrahimia canal (Table 2). On the other hand, 18 fungal taxa (*Anguillospora fustiformis, Anguillospora rosea*, *Condylospora gigantean, Cylindrocarpon aquaticum*, *Dactylella clavata*, *Dactylella cylindrospora, Dendroclathra lignicola*, *Dendrospora erecta*, *Filosporella aquatica, Flagellospora curvula, Leptodiscella brevicatenata, Lunulospora curvula*, *Mycocentrospora angulata*, *Mycocentrospora* sp., *Pseudoanguillospora stricta*, *Sympodioclathra globosa, Tumularia tuberculata, Varicosporium elodeae*, *Zopfiella longicaudata)* in addition to five unknown taxa appeared in El-Ibrahimia canal only. Micrographs of some isolated Aquatic Hyphomycetes from El-Zinnar and El-Ibrahimia canals were presented in (Fig. 3).

In this respect, El-Hissy et al*.* [29] collected 35 species related to 26 genera of aquatic hyphomycetes from submerged decaying leaves collected from various Egyptian water areas. Moreover, Khallil et al*.* [30] gathered 26 species assigning to 19 genera from water and submerged decaying leaves samples collected monthly in Egypt. Graca [41] collected only 12 aquatic fungal taxa from a river receiving strong mine and sewage pollution in Portugal. In Libya, Khallil [27] gathered 13 species of aquatic hyphomycetes from the rivulets of three hot springs. In Poland, Pietryczuk et al*.* [19] gathered 23 Ingoldian fungal species from selected rivers of Central Europe, with various contaminations. Moro et al*.* [20] gathered 39 species from water and submerged mixed leaf litter samples from 22 waterfalls and rivers at Ilhabela State Park, municipality of Ilhabela, São Paulo State, Brazil. Fiuza et al., (2022) reported that Angulospora aquatica, Biflagellosporella amazonensis, Isthmotricladia laeensis and Lateriramulosa bi-inflata were new records for Brazil wherease, Variocladium rangiferinum and Tricladium fuscum were new records for the Neotropics; and Polylobatispora deltoidea was a new record for the Americas [21].

### Seasonal fluctuation of Ingoldian fungi

The obtained data (Table 3) revealed that water temperature represents the main factor affecting the seasonal fluctuation of aquatic hyphomycetes as well as the low or moderate temperature is more favorable for aquatic fungi. Whereas, the highest dominance of aquatic hyphomycetes recovered from El-Zinnar canal was recorded during autumn (0.167) and the lowest dominance was estimated during winter (0.045). The highest simpson and Shannon diversity indexes of aquatic hyphomycetes recovered from El-Zinnar canal was estimated during winter recording 0.945 and 3.142, respectively and the lowest simpson (0.833) and Shannon (1.936) diversity indexes was recorded during autumn. Furthermore, The highest simpson and Shannon diversity indexes of ingoldian fungi recovered from El-Ibrahimia canal was recorded during winter estimating 0.932 and 2.756, respectively and the lowest simpson (0.913) and Shannon (2.563) diversity was recorded during summer (Table 3). As well as the present data showed that some Ingoldian fungal taxa showed variable occurrence patterns mainly according to the sampling season. In this respect, 6 genera (*Amniculicola*, *Anguillospora, Dactylella, Flagellospora, Mycocentrospora* and *Zopfiella*) and eight unknown taxa were represented throughout the year (three seasons at least for each). On the other side, ten genera (*Arbusculna, Cyrenella, Dendroclathra, Excipularia, Hormiokrypsis, Lemonniera, Pestalotiopsis*, *Sympodioclathra*, *Tricladiopsis* and *Ypsilina*) and two unknown taxa occurred exclusively in winter. Eight genera (*Calcarispora*, *Condylospora*, *Dendrospora*, *Diplocladiella, Filosporella, Guanomyces*, *Polycladium* and *Pseudallescheria*) in addition to two unknown taxa occurred exclusively in spring. Four genera namely, *Cylindrocarpon, Globoconidiopsis, Leptodiscella*, *Tumularia* in addition to six unknown taxa occurred exclusively in summer. *Brachiosphaera, Pseudoanguillospora, Tricladium* and two unknown taxa were represented in autumn only. Similar seasonal variations patterns were recorded for several Ingoldian fungal species (Table 2).

In agreement with our findings, Cudowski et al. [23] indicated that the lowest abundances of aquatic hyphomycetes were recorded during the summer season and the maximum abundance in the autumn. In contrast to our findings, the greatest diversity of Ingoldian fungi in the autumn compared to summer is due to autumnal shedding and falling of leaves from trees into the water areas, which provide the preferred substrates of aquatic hyphomycetes [42]. Ghate et al. [43], indicated that the total species diversity of Ingoldian fungi was higher in the wet season (lower temperature, lower conductivity and higher pH) than in the dry one.

### Correlation between abiotic factors of stressed water sites and species occurrence

**Fig. 4:**
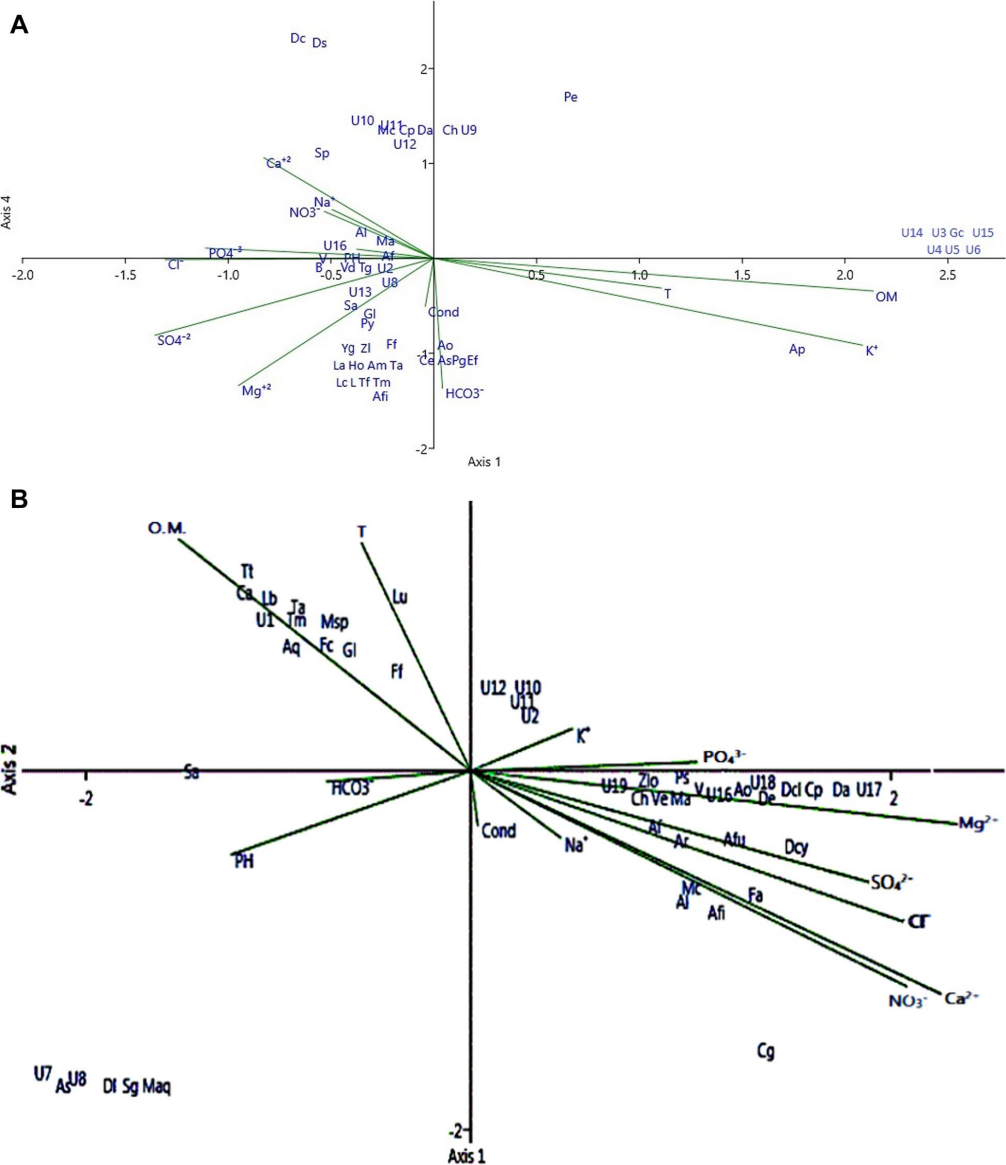
**A** The interaction of physicochemical parameters and ingoldian fungal composition in El-Zinnar irrigation canal in Arab El-Madabegh village. Al = *Amniculicola longissima*, Afi = *Anguillospora filiformis*, Af = *Anguillospora furtiva*, Ap = *Aquanectria penicillioides*, As = *Aquanectria submersa*, Am = *Arbusculna moniliformis*, Ao = *Arthrobotrys oligosporus*, B = *Brachiosphaera tropicalis*, Ch = *Calcarispora hiemalis*, Cp = *Curvularia pandanicola*, Ce = *Cyrenella elegans*, Da = *Dactylella arnaudii*, Dc = *Dactylella coccinella*, Ds = *Diplocladiella scalaroides*, Ef = *Excipularia fusispora*, Ff = *Flagellospora fusarioides*, Gl = *Glarea lozoyensis*, Gc = *Globoconidiopsis cerradensis*, Gp = *Guanomyces polythrix*, Ho = *Hormiokrypsis libocedri*, La = *Lemonniera aquatica*, Lc = *Lemonniera centrosphaera*, L = *Lemonniera cornuta*, Ma = *Mycocentrospora aquatica*, Mc = *Mycocentrospora clavata*, Pg = *Pestalotiopsis guepinii*, Pe = *Polycladium equiseti*, Pb = *Pseudallescheria boydii*, Py = *Pyramidospora casuarinae*, Sa = *Sigmoidea aurantiaca*, Sp = *Sirastachys phangngaensis*, Tf = *Tricladiopsis flagelliformis*, Tg = *Tricladium gracile*, Ta = *Triscelophorus acuminatus*, Tm = *Triscelophorus monosporus*, Vd = *Varicosporium delicatum*, V = *Varicosporium giganteum*, Yg = *Ypsilina graminea*, Zl = *Zopfiella latipes*, U2 = Unknown2, U3 = Unknown3, U4 = Unknown4, U5 = Unknown5, U6 = Unknown6, U8 = Unknown8, U9 = Unknown9, U10 = Unknown10, U11 = Unknown11, U12 = Unknown12, U13 = Unknown13, U14 = Unknown14, U15 = Unknown15, U16 = Unknown16. **B** The interaction of physicochemical parameters and ingoldian fungal composition in El-Ibrahimia canal affected by industrial effluents in Bani-Qurra village, Assuit, Egypt. Al = *Amniculicola longissima*, Afi = *Anguillospora filiformis*, Af = *Anguillospora furtiva*, Afu = *Anguillospora fustiformis*, Ar = *Anguillospora rosea*, Aq = *Aquanectria penicillioides*, As = *Aquanectria submersa*, Ao = *Arthrobotrys oligosporus*, Ch = *Calcarispora hiemalis*, Cg = *Condylospora gigantean*, Cp = *Curvularia pandanicola*, Ca = *Cylindrocarpon aquaticum*, Da = *Dactylella arnaudii*, Dcl = *Dactylella clavata*, Dcy = *Dactylella cylindrospora*, Dl = *Dendroclathra lignicola*, De = *Dendrospora erecta*, Fa = *Filosporella aquatica*, Fc = *Flagellospora curvula*, Ff = *Flagellospora fusarioides*, Gl = *Glarea lozoyensis*, Lb = *Leptodiscella brevicatenata*, Lu = *Lunulospora curvula*, Ma = *Mycocentrospora angulata*, Maq = *Mycocentrospora aquatica*, Mc = *Mycocentrospora clavata*, Msp = *Mycocentrospora* sp., Ps = *Pseudoanguillospora stricta*, Sa = *Sigmoidea aurantiaca*, Sg = *Sympodioclathra globosa*, Ta = *Triscelophorus acuminatus*, Tm = *Triscelophorus monosporus*, Tt = *Tumularia tuberculata*, Ve = *Varicosporium elodeae*, V = *Varicosporium giganteum*, Zlo = *Zopfiella longicaudata*, U1 = Unknown1, U2 = Unknown2, U7 = Unknown7, U8 = Unknown8, U10 = Unknown10, U11 = Unknown11, U12 = Unknown12, U16 = Unknown16, U17 = Unknown17, U18 = Unknown18, U19 = Unknown19

Canonical correspondence analysis demonstrated that the measured physicochemical properties remarkably affected the occurrence of Ingoldian fungal taxa in both water bodies (Fig. 4 A, B). The physicochemical parameters of surface water samples collected from El-Zinnar canal (Fig. 4 A) revealed that temperature and pH had influence on the occurrence of almost 60% of aquatic fungi recorded in this site, these fungi were *Anguillospora filiformis*,* Anguillospora furtiva*, *Aquanectria penicillioides*, *Aquanectria submersa*, *Arbusculna moniliformis*, *Brachiosphaera tropicalis*, *Calcarispora hiemalis*,* Curvularia pandanicola*, *Dactylella arnaudii*, *Dactylella coccinella*, *Diplocladiella scalaroides*, *Glarea lozoyensis, Guanomyces polythrix, Hormiokrypsis libocedri*, *Lemonniera aquatica*, *Lemonniera centrosphaera*, *Lemonniera cornuta*, *Mycocentrospora aquatica*, *Mycocentrospora clavata*, *Pestalotiopsis guepinii*, *Polycladium equiseti*, *Sirastachys phangngaensis, Tricladiopsis flagelliformis*, *Tricladium gracile*, *Varicosporium delicatum*, *Ypsilina graminea*, *Zopfiella latipes*, Unknown 8*,* Unknown10, Unknown11 and Unknown13, whereas *Amniculicola longissima*, *Cyrenella elegans*, *Excipularia fusispora*, *Flagellospora fusarioides*, *Pyramidospora casuarinae*, *Sigmoidea aurantiaca*, *Triscelophorus acuminatus*,* Triscelophorus monosporus*, *Varicosporium giganteum*, Unknown2, Unknown9, Unknown12 and Unknown16 showed clear relation to Mg^+2^ and pH. Temperature and potassium interacted with the frequency of Unknown3, Unknown4, Unknown5, Unknown6, Unknown14, Unknown15 and* Globoconidiopsis cerradensis,* but *Pseudallescheria boydii* and *Arthrobotrys oligosporus* depend on other studied physicochemical factors in their occurrence. Moreover, the relationship between physicochemical parameters of surface water samples collected from El-Ibrahimia canal water samples (Fig. 4 B) and occurrence of recovered aquatic ascomycetes and ingoldian fungal species revealed that HCO_3_^−^, K^+^, organic matter and temperature have interacted directly with the dominance of *Aquanectria penicillioides*, *Cylindrocarpon aquaticum*,* Leptodiscella brevicatenata*, *Flagellospora curvula, Flagellospora fusarioides, Glarea lozoyensis, Lunulospora curvula*,* Mycocentrospora* species,* Sigmoidea aurantiaca*,* Tumularia tuberculata*,* Triscelophorus acuminatus*, *Triscelophorus monosporus,* Unknown1, Unknown2, Unknown10, Unknown11 and Unknown12. On the other hand, the dissolved ions (Na^+^, Ca^+2^, Mg^+2^, Cl^−^, NO_3_^−^, SO_4_^−2^ and PO_4_^−3^) showed a relationship with the occurrence of aquatic fungal species *Amniculicola longissima*, *Anguillospora filiformis, Anguillospora furtiva*, *Anguillospora fustiformis, Anguillospora rosea*, *Arthrobotrys oligosporus*, *Calcarispora hiemalis, Curvularia pandanicola*, *Dactylella arnaudii*, *Dactylella clavata*, *Dactylella cylindrospora, Dendrospora erecta*, *Filosporella aquatica, Mycocentrospora angulata*, *Mycocentrospora clavata*, *Pseudoanguillospora stricta*, *Varicosporium elodeae*, *Varicosporium giganteum*, *Zopfiella longicaudata*, Unknown16, Unknown17, Unknown18 and Unknown19, Whereas pH, conductivity and Na^+^ related to the occurrence of *Aquanectria submersa*, *Condylospora gigantean, Dendroclathra lignicola*, *Mycocentrospora aquatica*, *Sympodioclathra globosa,* Unknown7 and Unknown8*.*

**Fig. 5:**
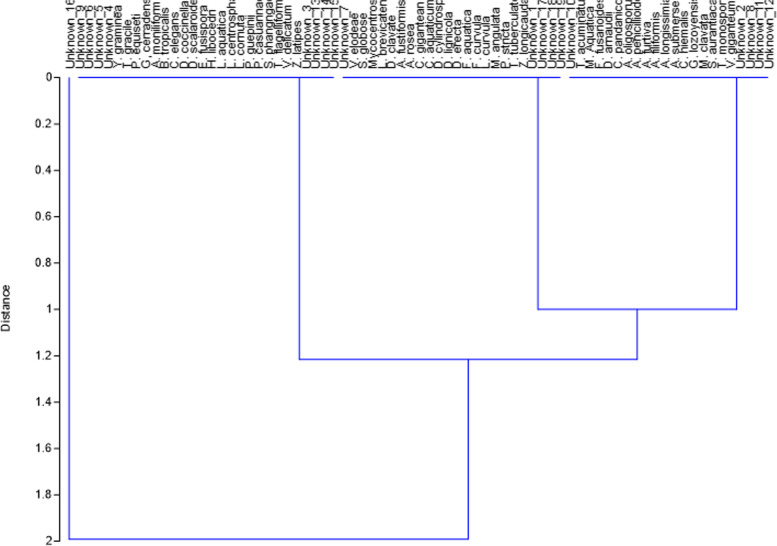
Cluster analysis showing the fungal communities recovered from El-Zinnar and El-Ibrahimia canals

Furthermore, the cluster analysis of aquatic hyphomycetes showed obvious grouping of the fungal communities; three separated fungal communities were presented (Fig. 5) which indicates that the different aquatic ecosystem types may showed a varied fungal community structure. Interestingly, the obtained data revealed that**,** the stressed water sites (ZN_3_ or IB_3_) which exposed directly to the effluents were frequently the poorest in fungal diversity, frequency of occurrence and abundance. These water sites can be regarded as extreme or stressful environments in which the ranges of one or more abiotic parameters are excessive of what would be considered normal for the growth and survival of ingolian fungi. It is concluded that occurrence and diversity aquatic hyphomycetes were conspicuously declined in these stressed water sites in comparison with the other less polluted sites. It is noteworthy and interesting to isolate some aquatic hyphomycetes from these stressful water sites which were characterized by relatively higher values of organic matter, water conductivity as well as cations and anions. In this respect, *Guanomyces polythrix,* *Pseudallescheria boydii* and *Aquanectria Penicillioides* in addition to two unknown taxa were emerged from the stressed water sites (ZN_3_ and ZN_4_) in El-Zinnar canal. Moreover, *Calcarispora hiemalis, Dendrospora erecta, Dactylella cylindrospora, Mycocentrospora angulata, Varicosporium elodeae* and three unknown taxa were isolated from the polluted water site (IB_3_) receiving the effluent of oils and detergents factory (El-Ibrahimia canal). Furthermore, the most predominant species were *Anguillospora furtive*, and *Amniculicola longissima* exhibiting the highest frequency of occurrence among the identified species and also emerged from both experimented water bodies irrespective to the sampling season. The remaining species were moderate, low or rare frequency of occurrence. Variable observations were recorded by many authors in different climatic regions worldwide. In this respect Khallil et al*.* [30] recorded that *Alatospora acuminate* and* Trisclophorus monosporus* were the most prevalent species in Egypt. Similar results obtained by Abdel-Raheem [44] who recorded that *Alatospora acuminata, Triscelophorus monosporus* and *Tetracladium marchalianum* were found to be the major colonizers on all leaf materials. In this respect, Meerbergen et al. [45] recorded that microbial composition and richness were lower in activated sludge from textile wastewater treatment plants compared to municipal wastewater treatment plants in Flanders (Belgium). However, Miransari [46] stated that fungi can help mitigate the adverse effects of various compounds by diverse mechanisms, including the production of organic products, chelating, precipitating or binding to heavy metals, retention and immobilization, or absorption of various pollutants such as heavy metals or dyes [47]. Our results support and corroborate the findings of Pietryczuk et al. [19] who revealed that water pollution usually leads to a decrease in the taxonomic diversity of aquatic hyphomycetes, including the complete disappearance of some species such as *Tetracladium breve* or *Tricladium* sp. Similarly, Selvarajan et al. [48] reported lower fungal diversity and species richness in highly contaminated environments.

In the other hand, It was reported that, pH always contributed a major explanation to the shaping of the fungal communities in a full-scale membrane bioreactor (MBR) for urban wastewater treatment [49]. Ortiz-Vera et al*.* [50] stated that pH facilitates bacterial growth, causing greater oxygen demand, decreasing the available oxygen in the water and consequently aquatic hyphomycetes. Lower pH is known to select for acid-tolerant fungi and to reduce overall fungal diversity increasing. Bai et al. [51] indicated that of the 11 environmental variables, organic matter, total nitrogen, water temperature, and pH were the dominant factors affecting fungal community composition. Abdel-Raheem et al. [52] concluded that pH value particularly plays a crucial role in determining the fungal diversity and their composition. On incongruity with our results, Pietryczuk et al. [19] revealed that the highest taxonomic diversity was determined for the waters with high organic matter concentrations, which simultaneously recorded the highest electric conductivity, concentrations of sulphate and chloride ions, total inorganic nitrogen, dissolved reactive phosphorus, constituting an indicator of water pollution of anthropogenic and agricultural origin while the lowest diversity for rivers poor in organic matter. Abdel-Raheem et al. [52] revealed that species composition was correlated positively with elevated concentrations of NH^3+^, calcium and magnesium have an inverse correlation on fungal diversity, no significant correlation of SO4^2−^ and NO^2−^ with the distribution of Ingoldian fungi but ammonia and phosphate encouraged fungal growth. Krauss et al. [53] concluded that the occurrence of *Heliscus lugdunensis* correlated with high concentrations of sulphate, nitrate, on contrast with the findings by Pietryczuk et al. [19] who reported that the occurrence of *Heliscus lugdunensis* is independent of the concentration of sulphate and total inorganic nitrogen. A positive correlation was recorded between *H. lugdunensis* and *A. longissima*, and the contents of organic matter [23, 54]. These findings indicate that these species may be reliable indicators of water pollution. Pietryczuk et al*.* [19] recorded that *Anguillospora crassa, Lunulospora curvula, Tetracladium breve, Tetracladium furcatum, Tricladium angulatum, Tricladium patulum, Tricladium splendens, Varicosporium delicatum*, and *Volucrispora graminea*, were found to be negatively correlated with both total inorganic nitrogen, sulphate and chloride ions. Therefore, they seem to be species preferring clean waters or waters with a moderate degree of pollution.

## Conclusion

The seasonality and distribution patterns of aquatic fungi in streams can be profoundly altered by different human activities, including the input of nutrients, untreated or semi-treated domestic wastes and sewage, various industrial wastes and pesticides. It seems that some Ingoldian fungal species can be a useful indicator of water quality, cleanliness and sanitary safety of surface waters, particularly in environmentally valuable areas. Our preliminary results demonstrate that some aquatic hyphomycetes are promising candidates in attempts to alleviate environmental pollution by stimulating native fungal populations. Further investigations should be conducted and hopefully coming in our Lab to reveal if Ingoldian fungi do accumulate toxic compounds and how decomposition rates are affected by pollutants.

## Data Availability

All the obtained data are included in the manuscript.
